# Posterior versus direct anterior approach in total hip arthroplasty: difference in patient-reported outcomes measured with the Forgotten Joint Score-12

**DOI:** 10.1051/sicotj/2018051

**Published:** 2018-11-27

**Authors:** Yu Ozaki, Tomonori Baba, Yasuhiro Homma, Hironori Ochi, Taiji Watari, Sammy Banno, Mikio Matsumoto, Kazuo Kaneko

**Affiliations:** Department of Orthopedic Surgery, Juntendo University School of Medicine, Tokyo Japan

**Keywords:** Total hip arthroplasty, Direct anterior approach, Posterior approach, Forgotten Joint Score-12.

## Abstract

*Introduction*: When the postoperative outcome of primary total hip arthroplasty (THA) was compared with the direct anterior approach (DAA) and the posterior approach (PA), there was no significant difference of the clinical outcome at 6 months to 1 year after surgery in many studies. This study was performed to compare the medium-term outcome of THA via the DAA or PA and clarify which approach achieves better quality of life (QOL).

*Methods*: We investigated 61 hips receiving primary THA (30 via DAA and 31 via PA), using hip function scores such as the Harris Hip Score (HHS) and patient-reported outcomes such as the Western Ontario and McMaster Universities Osteoarthritis Index (WOMAC), the Japanese Orthopaedic Association Hip Disease Evaluation Questionnaire (JHEQ), and the Forgotten Joint Score-12 (FJS).

*Results*: The mean duration of postoperative follow-up was 36.8 months in the DAA group and 40.5 months in the PA group. There was no difference in preoperative or postoperative HHS between the two groups. Although there was no difference of postoperative WOMAC and JHEQ, the postoperative FJS-12 score was significantly higher in the DAA group than in the PA group (75.2 ± 15.9 versus 60.1 ± 24.4, *p* = 0.01).

*Conclusion*: When forgetting the artificial joint in daily life is the target, better QOL can be achieved by performing THA via the DAA.

## Introduction

Total hip arthroplasty (THA) is an effective surgical procedure that leads to pain relief and functional recovery of the hip joint with improvement in the quality of life (QOL) [1]. There are several approaches for primary THA, and selection of the approach that achieves the most favorable postoperative outcome is still controversial.

The posterior approach (PA) is most widely employed for THA, since it provides adequate visualization of the hip joint and surrounding soft tissues, along with superior versatility and operability [2]. On the other hand, the direct anterior approach (DAA) has recently been attracting attention as the only approach employing an intermuscular and internervous plane [3]. It has advantages for pain relief and functional recovery early after surgery [4,5]. Although there have been reports that the PA is associated with a slightly higher dislocation rate compared with other approaches [6,7], it is considered that the dislocation rate can be reduced by soft tissue repair [7,8]. It has also been reported that there is no difference in the dislocation rate between the PA and DAA [9].

When the postoperative outcome of primary THA was compared between the PA and DAA [10–14], some authors found that pain relief early after surgery was greater and functional recovery was faster when the DAA was used [15,16], while others found no significant difference of clinical outcomes from 6 months to 1 year after surgery [10,11]. However, the postoperative follow-up period was less than 1 year in most of these studies, and there have been few comparisons of the two approaches beyond 1 year.

We hypothesized that use of the DAA (muscle-sparing approach) would achieve better clinical outcomes than the PA if the follow-up period was longer. Therefore, this study was performed to compare the postoperative medium-term outcome of THA between the DAA and PA using patient-reported outcomes (PROs), in order to clarify which approach achieves better QOL after surgery.

## Materials and methods

**Figure 1:**
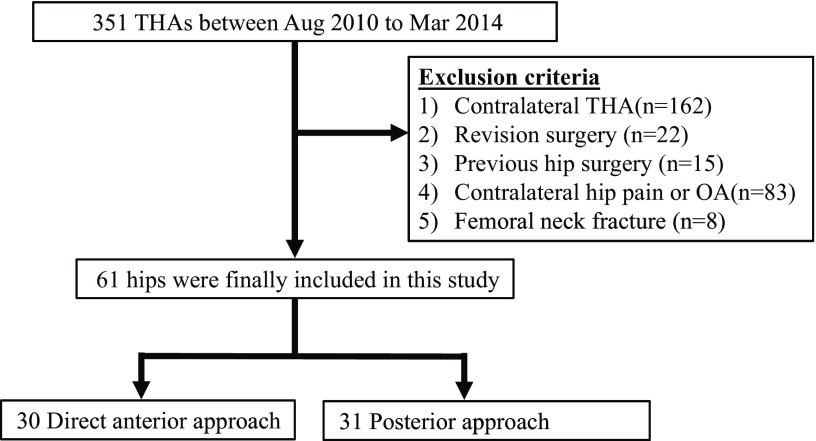
Flow chart of this study.

Of 351 patients undergoing THA between August 2010 and March 2014, 61 patients who received unilateral primary THA were included. Surgery was performed by the PA until October 2012 and by the DAA from November 2012. We excluded patients with a history of previous surgery on the affected hip or surgical repair of femoral neck fracture (Figure 1). Because it has been reported that the contralateral hip influences patient-reported outcomes [12], we also excluded patients with contralateral THA and pain or arthropathy of the contralateral hip joint. Surgery was performed via the PA in 31 patients (6 males and 25 females) and via the DAA in 30 patients (5 males and 25 females). The same four hip surgeons performed the operations by both approaches.

## Surgical procedures

Uncemented THA was performed by four surgeons using either the DAA or the PA according to the standard methods for these widely used approaches. A brief description of the two procedures is provided below.

THA via the DAA was performed by all four hip surgeons using the same surgical protocol with the patient in the supine position on a standard operating table. The skin incision was started 2 cm lateral and distal to the anterior superior iliac spine and was extended distally for up to 10 cm along a line angling toward the head of the fibula. Briefly, the fascia of the tensor fascia latae (TFL) muscle was incised about 2 cm lateral to the skin incision to prevent lateral femoral cutaneous nerve injury, and the intermuscular space between the TFL and sartorius muscles was entered by blunt dissection. The anterior articular capsule was exposed and incised as widely as possible to expose the femoral head. For stem insertion, the operating table was extended so that the hip joint could be extended to 15°. The superior and posterior portions of the articular capsule were partially incised so that the greater trochanter could be elevated with a retractor.

THA via the PA was also performed by all four hip surgeons using the same surgical protocol with a standard operating table and the patient in the lateral decubitus position. A 10–12 cm skin incision was centered over the posterior aspect of the great trochanter. The gluteus maximus muscle was split along the line of its fibers. The short external rotators and the posterior capsule were incised. Then the hip was dislocated posteriorly and femoral neck osteotomy was performed. After insertion of the acetabular and femoral components, the short external rotators and posterior capsule were repaired.

All patients received standardized postoperative treatment, including pain management and rehabilitation. Physical therapy was initiated on postoperative day one or two, depending on the patient's immediate postoperative recovery and clinical condition. All patients were allowed to perform full weight bearing to tolerance, progressing from a walker to a cane to no assistance as tolerated. Patients undergoing THA by either approach were instructed not to hyperflex, adduct, and internally rotate the lower limb. Patients undergoing THA by the DAA were also instructed about extension and external rotation of the lower limb. Patients were discharged from hospital when able to safely mobilize for performance of daily activities.

## Outcome measures

### Patient-reported outcomes (PROs)

Outcomes were assessed by using the Western Ontario and McMaster Universities Osteoarthritis Index (WOMAC) (pain, stiffness, and function subscales), the Japanese Orthopaedic Association (JOA) Hip Disease Evaluation Questionnaire (JHEQ) [13], and the Forgotten Joint Score-12 (FJS-12) [14]. The WOMAC was first reported by Bellamy and Buchanan in 1986, and is used worldwide to evaluate the lower limbs, particularly the hip and knee joints [total score ranges from 0 (best) to 96 (worst)]. The JHEQ is a validated self-administered questionnaire for evaluating the QOL of patients with hip disease and an Asian lifestyle [total score ranges from 0 (worst) to 84 (best)] [13]. The FJS-12 is another self-administered questionnaire, which is based on the concept that the ultimate goal of THA is for patients to forget they have an artificial joint, and is reportedly a useful tool for assessing PROs specific to artificial joints [14]. The FJS-12 is a specific and subjective PRO tool that assesses patient awareness of the artificial knee or hip joint during activities of daily living [total score ranges from 0 (worst) to 100 (best)] [14]. These questionnaires were mailed to the patients, who filled in the answers and then mailed them back to our department.

To assess hip function, the Harris Hip Score (HHS) was determined at an outpatient consultation near the time when the PRO questionnaires were sent and just before surgery. The percentage of patients with no limitation of the walking distance, the ability to navigate stairs normally, or the ability to put on shoes and socks easily was determined, and the range of flexion, abduction, adduction, internal rotation, and external rotation were evaluated.

### Statistical analysis

The scores indicating the best results are 0 for the WOMAC and 84 for the JHEQ. Therefore, the lowest and highest scores for each questionnaire were converted to 0 and 100, respectively.

To assess the impact on patient QOL and hip function, we used the Mann–Whitney U test to compare differences of the PRO questionnaires and the HHS between the two approaches. Demographic data were compared by using Fisher's exact test for categorical variables and the *t*-test for numerical variables. The percentages of subjects with no limitation of the walking distance, the ability to navigate stairs normally, and the ability to put on shoes and socks easily were evaluated by the HHS and compared using Fisher's exact test [16]. In all tests, a *p* value less than 0.05 was considered statistically significant.

## Results

The mean duration of follow-up after surgery was 36.8 months (range: 25–48 months) in the DAA group and 40.5 months (range: 28–50 months) in the PA group. Analysis of demographic data (Table 1) revealed no significant differences of the age, sex, body mass index, or laterality between the groups. There were also no significant differences of the operating time or blood loss between the groups. Revision surgery was not performed in either group.

**Table 1:** Demographic and surgical data.

Variables	Direct anterior approach	Posterior approach	*p* value
Age (years)	64.5 ± 9.7	66.3 ± 10.0	0.49
Sex (female, %)	83.3	80.6	0.52
Body mass index (kg/m^2^ )	23.5 ± 2.7	23.1 ± 4.7	0.68
Diagnosis of osteoarthritis (%)	83.3	90.3	0.33
Laterality (left hip, %)	53.3	54.8	0.55
Surgery time (min)	123.2 ± 21.3	116.9 ± 35.4	0.40
Blood loss (ml)	457 ± 233	478 ± 179	0.68

The most used Femoral head size was 32 mm head (19 of 30 patients) in the DAA group and 28 mm head (17 of 31 patients) in the PA group. For femoral head material, metal head was used more in the PA group (28 of 31 patients). In the DAA group, 6 of 30 patients used dual-mobility bearings (Table 2).

**Table 2:** Implants used in DAA and PA groups.

Implant	Type	Direct anterior approach (*n* = 30)	Posterior approach (*n* = 31)
Femoral head size (mm)	22	1	0
	28	5	17
	32	19	13
	36	5	1
Bearings	Fixed-bearings	24	31
	Dual-mobility bearings	6	0
Acetabular liner	HXLPE	30	31
Femoral head material	Head, ceramic	12	3
	Head, metal	18	28

HXLPE: highly cross-linked polyethylene

Regarding hip function (Table 3), there was no significant difference of the preoperative HHS between the two groups. Postoperatively, the two groups did not differ significantly with regard to the proportion of patients with no limitation on walking distance, the ability to navigate stairs normally, or the ability to put on shoes and socks easily. No significant difference of the total HHS was noted between the groups.

**Table 3:** Harris hip score of before surgery and at 3 years after surgery.

Variables	Direct anterior approach (*n *= 30)	Posterior approach (*n *= 31)	*p* value
*Preoperative*			
Total	50.7 ± 10.6	54.9 ± 10.2	0.12
*Postoperative*			
Unlimited walking (%)	76.7	80.6	0.47
Stairs normally (%)	83.3	87.0	0.48
Shoes and socks with ease (%)	80.0	67.7	0.21
Total	91.3 ± 4.5	90.5 ± 6.4	0.76

Perioperative complications occurred in seven patients from the DAA group and two patients from the PA group. In the DAA group, the complications were lateral femoral cutaneous nerve injury in seven patients. Conservative treatment was selected in all cases. Dislocation occurred in two patients from the PA group. It was treated with closed reduction, and did not recur. Regarding PROs (Table 4), the FJS-12 score was significantly higher in the DAA group than in the PA group (75.2 ± 15.9 versus 60.1 ± 24.4, *p* = 0.01). However, there were no significant differences of the JHEQ score or the WOMAC score between the groups (81.6 ± 17.3 versus 74.4 ± 16.2, *p* = 0.05, 91.7 ± 12.6 versus 90.7 ± 10.5, *p* = 0.47).

**Table 4:** Patient-reported outcomes at 3 years after surgery.

Variables	Direct anterior approach (*n* = 30)	Posterior approach (*n* = 31)	*p* value
WOMAC	91.7 ± 12.6	90.7 ± 10.5	0.47
JHEQ	81.6 ± 17.3	74.4 ± 16.2	0.05
FJS-12	75.2 ± 15.9	60.1± 24.4	0.01

WOMAC: Western Ontario and McMaster Universities Osteoarthritis Index, JHEQ: JOA Hip Disease Evaluation Questionnaire, FJS-12: Forgotten Joint Score-12).

## Discussion

Many authors have reported that differences in the clinical outcome of primary THA via the PA or DAA were undetectable by 6–12 months after surgery [10,11]. However, the present study followed patients for 3 years after surgery and revealed that the FJS-12 score was significantly higher in DAA group than in the PA group. This suggests that if forgetting the artificial joint in daily life is set at the target, better QOL can be achieved by performing THA via the DAA.

The FJS-12 is intended to determine “whether one can live while forgetting the presence of an artificial joint” [14]. It evaluates whether patients are “aware of” the artificial joint during various daily activities. For example, “Awareness when you are walking for more than 15 min?” “Awareness climbing stairs?”  “Awareness when standing up from a low-sitting position? ” Even if the patients are painless and these hip joint function is improved, the score of FJS-12 will be lower if patients are “aware of” the artificial joint in various motions of daily life. Thus, slight complaints not identified by a specific question such as “Can you do the motion?” are picked up as “being aware of” the joint, which may decrease the ceiling effect and more sensitively reflect QOL after surgery [14,17].

We consider that the main factor that influences forgetting the artificial joint in everyday life is hip joint stability during movements related to daily activities. Although the functional outcome was comparable between the DAA and PA groups, the FJS-12 revealed a significant difference in the present study. This suggests that even though they could perform a certain motion, patients who underwent THA via the PA were aware of the prosthesis during motion. We think that the main factor underlying this difference is the posterior soft tissues.

The posterior soft tissues contribute to hip joint stability [16], and damage to these tissues may have influenced “awareness” in the FJS-12, although no difference was observed with other tools such as the WOMAC or HHS. We believed that the FJS-12 is the tool that can express the “stability feeling” as “aware of”. Ranawat et al. performed magnetic resonance imaging (MRI) at 2 years after THA via the PA and observed moderate to severe atrophy of the repaired short external rotators and incomplete healing of tendons in about 60% of the patients [18]. In addition, McLawhorn et al. performed MRI evaluation more than 4 years after THA via the PA [19]. Regarding soft tissue repair after transection, they reported scar tissue between the piriformis and conjoined tendons, with bone remodeling to achieve an orientation and MRI signal intensity resembling the native tendon in the majority of patients [19]. No matter how the transected structures are restored, the posterior tissues may be weaker than before surgery. Barrett et al. mentioned that preservation of muscle attachments to bone and avoidance of muscle division offer the potential for improved dynamic hip stability [16].

When THA is performed via the DAA, the gluteus maximus is not incised. Nakata et al. stated that the gluteus maximus muscle is very important for hip extension and for activities such as getting out of a chair, going up and down stairs, and getting out of a car [15]. In addition, the gluteus maximus and tensor fascia latae are inserted into the iliotibial band to form a “deltoid of the hip” involved in pelvic stabilization, and this function is undisturbed when the DAA is employed. The short external rotators also play an important role in hip function and dynamic hip stabilization. When using the DAA, exposure of the femoral medullary canal is often difficult and may require posterolateral release, with the subsequent risk of releasing the external rotators [20,21]. In a cadaver study, Meneghini et al. found that the piriformis or conjoint tendon was transected in 50% of patients who had undergone THA via the DAA [20]. However, another cadaver study by Oldenrijk et al. showed that THA via the DAA could better preserve the piriformis compared with other approaches for minimally invasive surgery [21]. Kawasaki et al. performed MRI evaluation at 1 year after THA via the DAA and reported that there was no significant change of the piriformis compared with before surgery [22]. Postoperative MRI evaluation by Cristoph et al. revealed that damage to the external rotator tendons was markedly more severe with the PA compared to other approaches including the DAA [23]. We think that “stability feeling” in motions of daily life depends on the degree of damage of the posterior soft tissues, which is the dynamic stabilizer. Although the DAA can also lead to damage to the posterior muscles, the dynamic hip stability may be better maintained after THA via the DAA than the PA. Therefore, we consider that patients in the DAA group were more able to forget about the artificial joint due to better “stability feeling” compared with the PA group.

The duration of postoperative follow-up was 3 years in this study. There was a significant difference in the FJS-12 score between the DAA and PA groups at 3 years, but no difference in the more frequently used WOMAC score. This suggests that the FJS-12 is an appropriate tool for evaluating QOL both early after surgery and also in the medium-term when the WOMAC cannot detect differences. we believe that FJS − 12 can reflect slight complains of patient's QOL in the medium and long term sensitively.

Lateral femoral cutaneous nerve injury is a characteristic complication of DAA. We have reported that this complication can lead to awareness of the prosthesis after surgery, influencing the FJS-12 score [24]. Lateral femoral cutaneous nerve injury was observed in seven patients from the DAA group. Although this number is not small, we have previously demonstrated that lateral femoral cutaneous nerve injury mainly resolves spontaneously with time after THA via the DAA [25]. If the influence of nerve injury on the FJS-12 decreases over the time, this may also be a factor leading to a better FJS score at 3 years after surgery in the DAA group.

Several limitations of this study should be considered. First, the number of patients was small in both groups due to exclusion of patients with THA or osteoarthritis of the contralateral hip joint. However, patients were selected carefully because contralateral hip joint pain, limitation of the range of motion, or the presence of a prosthetic joint may act as confounding factors, since pathology of the contralateral hip joint has been reported to influence patient-reported evaluations [12]. Second, THA was performed by multiple surgeons with different levels of experience in both the DAA group and the PA group. However, the same operative procedure was used and all of the surgeons were in a single team. Third, the ratio of used bearing and femoral head size is different between the two groups. However, it is reported that there is no significant difference in hip joint function and PROs depending on the size of femoral head size and difference in the type of bearings including fixed bearings or dual-mobility bearings [26–28]. Fourth, we have investigated FJS-12 only 3 years after surgery. The significant differences may appear even in a shorter period of time. Therefore, we consider that a prospective survey as of the half year, 1 year, and 2 years is necessary in the future.

## Conflict of interest

The authors declare that they have no conflict of interest in relation to this article.
